# Medium-chain fatty acids decrease serum cholesterol via reduction of intestinal bile acid reabsorption in C57BL/6J mice

**DOI:** 10.1186/s12986-018-0267-x

**Published:** 2018-06-05

**Authors:** Huizi Li, Yinghua Liu, Xinsheng Zhang, Qing Xu, Yong Zhang, Changyong Xue, Changjiang Guo

**Affiliations:** 1 Department of Nutrition, Tianjin Institute of Environmental & Operational Medicine, Tianjin, 300050 China; 2 Department of Nutrition, PLA Rocket Force General Hospital, Beijing, 100088 China; 3 Department of Nutrition, Chinese PLA General Hospital, Beijing, 100853 China

**Keywords:** Medium-chain triglyceride, Medium-chain fatty acids, Bile acids, Ileal bile acid binding protein, Caco-2 cells

## Abstract

**Background:**

Bile acids play a pivotal role in cholesterol metabolism via the enterohepatic circulation. This study investigated the effects of medium-chain triglycerides (MCTs)/medium-chain fatty acids (MCFAs) on the reduction of bile acid absorption in the small intestine and the mechanisms of action in vivo and partially verified in vitro.

**Methods:**

Thirty-six C57BL/6 J mice with hypercholesterolaemia were randomly divided into 3 groups: fed a cholesterol-rich diet (CR group), fed a cholesterol-rich and medium-chain triglyceride diet (CR-MCT group) and fed a cholesterol-rich and long-chain triglyceride diet (CR-LCT group). Body weights and blood lipid profiles were measured in all groups after 16 weeks of treatment. The concentrations of bile acids in bile and faeces were analysed using HPLC-MS (high-performance liquid chromatography-mass spectrometry). Gene transcription and the expression levels associated with bile acid absorption in the small intestines were determined using real-time PCR and Western blot. Ileal bile acid binding protein (I-BABP) was analysed using immunofluorescence. The effects of MCFAs on the permeability of bile acid (cholic acid, CA) in Caco-2 cell monolayers and I-BABP expression levels in Caco-2 cells treated with caprylic acid (C8:0), capric acid (C10:0), stearic acid (C18:0) and oleic acid (C18:1) were determined.

**Results:**

Mice in the CR-MCT group exhibited lower body weights and serum total cholesterol (TC) and low-density lipoprotein cholesterol (LDL-C) levels and a higher HDL-C/LDL-C ratio than the CR-LCT group (*P* < 0.05). The concentrations of primary bile acids (primarily CA) and secondary bile acids in faeces and secondary bile acids in bile in the CR-MCT group were significantly higher than in the CR-LCT group (*P* < 0.05). C8:0 and C10:0 decreased the permeability of CA in Caco-2 cell monolayers. MCT/MCFAs (C8:0 and C10:0) inhibited I-BABP gene expression in the small intestines and Caco-2 cells (*P* < 0.05).

**Conclusions:**

MCT slowed the body weight increase and promoted the excretion of bile acids. MCT lowered serum cholesterol levels at least partially via reduction of bile acid absorption in the small intestine by inhibition of I-BABP expression. Our results provide the basis for clinical trials of MCT as a dietary supplement for lowering plasma cholesterol and reducing risk of CHD.

## Background

The latest data from the World Health Organization reveal that cardiovascular diseases (CVDs), principally stroke and coronary heart disease (CHD), remain the major cause of human death worldwide and claim the lives of 17.7 million people annually (31% of all global deaths) (http://www.who.int/cardiovascular_diseases/en/). High blood cholesterol is a crucial risk factor for CVDs [1]. A projected increase of serum total cholesterol (TC) increase of 0.58 mmol/L (22.4 mg/dL) in Chinese men and 0.55 mmol/L (21.6 mg/dL) in Chinese women from 2010 to 2030 would produce the highest increase in CHD events (increase by 31% of CHD baseline events in men and 15% in women) of all risk factors modeled in China [2].

Bile acids play a pivotal role in cholesterol metabolism via the enterohepatic circulation. Bile acids are converted from cholesterol in liver cells, and 90%–95% of bile acids are reabsorbed into the intestinal epithelial cells at the end of the ileum and transported to the blood circulation to re-enter liver cells. Only approximately 5% of bile acids in the intestine are excreted in the stool. The physiological significance of enterohepatic circulation of bile acids is to make the limited bile acid reusable for maintaining cholesterol homeostasis, and to facilitate the elimination of excess cholesterol from the body [3].

Medium-chain triglycerides (MCTs) with 8–12 carbon atoms is digested by lipase in the stomach and duodenum into glycerol and medium-chain fatty acids (MCFAs). Our previous studies confirmed that a diet containing MCFAs reduced serum total cholesterol (TC) via promotion of bile acid synthesis in the liver and increased excretion of bile acids in faeces [4, 5]. We hypothesized that the small intestine also plays an essential role in the maintenance of cholesterol balance. Therefore, the present study investigated whether MCT decreased serum cholesterol via inhibition of bile acid reabsorption in the small intestine and the expression of relevant transporters of bile acid reabsorption in the small intestine in C57BL/6 J mice and Caco-2 cell monolayers.

## Methods

### Animals and diets

Male C57BL/6 J mice (4 weeks old, *n* = 80) were purchased from the Institute of Laboratory Animal Science, Chinese Academy of Medical Sciences (License No. SCXK: JING2009–0007) and housed in a temperature-controlled room (22 ± 2 °C, 40% to 60% humidity) with a 12-h light/dark cycle. The Animal Care and Use Committee of the Chinese PLA General Hospital approved all experimental procedures.

Mice were fed a basal diet (AIN-93G diet, purchased from the Academy of Military Medical Science) during the first week for adaptation. Ten mice were randomly chosen and fed a basal diet as the normal control, and the remaining mice were fed a cholesterol-rich (CR) diet modified from the AIN-96G diet. Blood samples were collected from the mandibular venous plexus two weeks later. Mice with serum TC levels that were 2 standard deviations greater than the normal control (accounting for 67% of mice fed the CR diet) were randomly assigned to three groups (*n* = 12 in each group). Each group was fed a different diet for 16 weeks (Table 1): cholesterol-rich and medium-chain triglyceride (CR-MCT), cholesterol-rich and long-chain triglyceride (CR-LCT) or CR. Nisshin Oillio (Tokyo, Japan) donated the MCTs (C8:0 and C10:0) and LCTs (long-chain triglycerides, soy bean oil). Cholesterol was purchased from Sigma-Aldrich (St. Louis, MO, USA). The Beijing Institute of Nutrition Resources (Beijing, China) measured the fatty acid compositions of the CR-MCT and CR-LCT diets (Table 2). Body weights and food intake were monitored weekly.

**Table 1:** Composition and nutrition factors of experimental diets for C57BL/6 J mice

Ingredients	CR-MCT	CR-LCT	CR
Basal diet (%)	86.7	86.7	88.7
Lard (%)	10	10	10
Cholesterol (%)	1	1	1
Bile salt (%)	0.3	0.3	0.3
MCT (%)	2	–	–
LCT (%)	–	2	–
Energy (KJ/g)	16.81	16.81	16.39
Percentage of Nutrients			
Protein (%)	18.69	18.69	19.07
Fat (%)	15.36	15.36	13.63
Carbohydrate (%)	47.22	47.22	48.18
Cholesterol (%)	1	1	1
Mineral mixture (%)	1.06	1.06	1.08
Vitamin mixture (%)	0.51	0.51	0.52
Fibre (%)	1.47	1.47	1.5
Water (%)	9.11	9.11	9.3
Others (%)	5.58	5.58	5.72

**Table 2:** Fatty acid composition of CR-MCT, CR-LCT and CR diets

Fatty acids	CR-MCT		CR-LCT C18:3		CR C18:0	
	g/100 g diet	%	g/100 g diet	%	g/100 g diet	%
C8:0	1.51	9.75	ND^a^	ND^a^	ND^a^	ND^a^
C10:0	0.55	3.55	ND^a^	ND^a^	ND^a^	ND^a^
C14:0	0.01	0.06	0.01	0.06	0.01	0.07
C16:0	3.09	19.95	3.51	22.52	3.13	22.90
C16:1	0.19	1.23	0.19	1.22	0.19	1.39
C17:0	0.06	0.39	0.06	0.39	0.06	0.44
C17:1	0.02	0.13	0.02	0.13	0.02	0.15
C18:0	1.47	9.49	1.66	10.68	1.54	11.27
C18:1	5.26	33.96	6.21	39.88	5.36	39.21
C18:2	3.09	19.95	3.61	23.16	3.12	22.82
C18:3	0.18	1.16	0.22	1.39	0.18	1.32
C20:0	0.03	0.19	0.04	0.26	0.03	0.22
C20:4	0.01	0.06	0.02	0.13	0.01	0.07
C20:5	0.01	0.06	0.02	0.13	0.01	0.07
C22:0	0.01	0.06	0.01	0.06	0.01	0.07
Total	15.49	100	15.58	100	13.67	100

^a^not detectable

### Blood, ileum, bile and faeces sampling

Five mice were chosen randomly from each group after 16 weeks of feeding to record diet intake and faeces excretion using separate metabolic cages for 3 days. Faeces were lyophilized, weighed, pulverised, and stored at − 80 °C for further analysis. All mice were fasted overnight (approximately 12 h) prior to blood sample collection from the aorta ventralis under anaesthesia (xylazine hydrochloride). Serum was collected after centrifugation of the blood samples. A microcapsule was used to puncture the gallbladder wall and extract the bile, and each bile sample was centrifuged at 16,000 g for 30 min. The supernatant was collected and stored at − 80 °C. Ileum tissues were excised and rinsed with ice-cold saline, and portions were immediately stored at − 80 °C for further analysis.

### Measurement of serum lipid profiles

Serum TC and triglyceride (TG) were determined at the end of the experiment using commercial kits from Wako (Osaka, Japan). High-density lipoprotein-cholesterol (HDL-C) and low-density lipoprotein-cholesterol (LDL-C) were measured using sediment methods and a commercial kit from Abcam (Cambridge, UK). Serum total bile acid (TBA) was measured using a commercial kit from Blue Gene (Shanghai, China). All manufacturer’s instructions were strictly followed.

### Analysis of bile acids in faeces and bile using HPLC-MS

The methods for the preparation and determination of bile acids in faeces and bile were performed according to our previous study and reports from Steiner et al. [4, 6]. A Shimadzu LC-20 AD high-performance liquid chromatography system (Shimadzu, Kyoto, Japan) coupled to an Applied Biosystecm 3200 Q TRAP mass spectrometer with an electrospray ionization (ESI) source (Applied Biosystems, Carlsbad, USA) was used. The HPLC and MS systems were controlled using Analyst 1.4.2 software. All chromatographic separations were performed using a Venusil AQ C18 column (2.5 μm, 50 mm × 2.1 mm) (Agela Techonologies, Delaware, USA). Cholic acid (CA), deoxycholic acid (DCA), chenodeoxycholic acid (CDCA), ursodeoxycholic acid (UDCA), lithocholic acid (LCA), taurocholic acid (TCA), taurodeoxycholic acid (TDCA), taurochenodeoxycholic acid (TCDCA), taurolithocholic acid (TLCA), glycocholic acid (GCA), glycodeoxycholic acid (GDCA) and glycochenodeoxycholic acid (GCDCA) were used as standard substances. Chloramphenicol was used as an internal standard. The abovementioned materials were purchased from Sigma-Aldrich (St. Louis, MO, USA). Primary bile acids, including CA, CDCA, TCA, TCDCA, GCA and GCDCA, and secondary bile acids, including DCA, LCA, UDCA, TLCA, TDCA and GDCA, in bile and faeces were determined by the retention times.

### Quantitative real-time PCR (qRT-PCR)

Samples from small intestine tissue (approximately 100 mg) were washed with ice-cold PBS and homogenized. Total RNA from tissues and cells was isolated using Trizol reagent (Omega Bio-Tek, Norcross, USA, no. R6812). Primers were designed using Primer Express 3.0 software based on the mRNA sequences from a database (Table 3) and synthesized by Invitrogen (Beijing, China). Methods for RNA extraction and quantitative real-time PCR (qRT-PCR) are described in our previous publications. qRT-PCR was performed using a One Step SYBR® PrimeScript® RT-PCR Kit (Takara Biotechnology Co., Ltd., Dalian, China). Amplification was performed using a BIO-RAD iCycler Thermal Cycler (BIO-RAD, Hercules, CA, USA). Relative mRNA expression levels were determined using the comparative critical threshold (Ct) method (in a separate tube). The housekeeping gene β-actin was used as a control for normalization.

**Table 3:** Primer sequences used for mRNA quantification with qRT-PCR

Target Genes	Human / Mouse	Forward (5′-3′)	Reverse (5′-3′)	Accession no.
ASBT^a^	M	AGGCTTTATCCTGTCTGTGGC	CAGTGTGGAGCAAGTGGTCAT	NM_011388.2
I-BABP^b^	H	GAGAGCTGTGTTGTCTGCGT	TTGAAGTTGCGGGCCTTTTC	NM_001040442.1
	M	GATCATCACAGAGGTCCAGC	CTCCATCTTCACGGTAGCCT	NM_008375.2
OST-α^c^	M	TCCCTGACGGCATCTATGAC	ACAAGCACCTGGAACAGAGC	NM_145932.3
OST-β^d^	M	GGAACTGCTGGAAGAAATGC	TTCTGTTTGCCAGGATGCTC	NM_178933.2

^a^gene name: a solute carrier family 10, member 2 (Slc10a2), protein name: apical sodium-dependent bile salt transporter (ASBT)

^b^gene name: fatty acid binding protein 6 (FABP6/fabp6), protein name: ileal bile acid binding protein (I-BABP)

^c^gene name: solute carrier family 51, alpha subunit (Slc51a), protein name: organic solute transporter (Ost-α)

^d^gene name: solute carrier family 51, beta subunit (Slc51b), protein name: organic solute transporter (Ost-β)

### Western blot assay

Western blot analyses of small intestine tissue and cultured cells were performed as described previously [7]. Equivalent amounts of protein from each sample were prepared and separated using SDS-PAGE (12% gels) followed by electrotransfer to PVDF membranes. Membranes were incubated with blocking solution (Tris-buffered saline, 8% non-fat dry milk) for 2 h followed by incubation with specific antibodies at 4 °C overnight. Membranes were washed extensively in TBST (Tris-buffered saline with Tween, containing 0.5% Tween-20 in TBS) and incubated with horseradish peroxidase-conjugated secondary antibodies in TBST for 1 h. Membranes were washed further in TBST, and bands were detected using chemiluminescence detection agents. Blot densitometry was performed, and the bands were analysed using ImageJ software. Antibodies targeting the apical sodium-dependent bile acid transporter (ASBT), ileal bile acid binding protein (I-BABP) and organic solute transporter α/β (Ostα/β) were purchased from Abcam (Cambridge, UK). Secondary antibodies against goat IgG were obtained from Sun Biomedical Technology Co. (Beijing, China).

### Immunofluorescence

Ileum tissue fixed with 4% buffered paraformaldehyde was embedded in paraffin, and 4-μm thick sections were prepared. Sections were deparaffinized and quenched in 3% H_2_O_2_ for 15 min to block endogenous peroxidase. Sections were washed in PBS and incubated with an anti-I-BABP antibody in PBS (1:50 dilution) for 1 h at 37 °C. A FITC-conjugated (fluorescein isothiocyanate) antibody was added at a 1:50 dilution and incubated for 0.5 h at 37 °C. Sections were washed, and PI (propidium iodide) was used to stain the nuclei. I-BABP was imaged using a fluorescence microscope (BX60, Olympus, Ina, Japan). I-BABP was observed as green fluorescence, and the nucleus was observed as red fluorescence [8].

### Caco-2 cell culture

The Caco-2 cell line was obtained from the Cell Resource Center, Peking Union Medical College (which is the headquarters of the National Infrastructure of Cell Line Resource) on Sept. 20, 2016. PCR and culture confirmed that the cell line was checked free of mycoplasma contamination. The species origin of these cells was confirmed using PCR. The identity of the cell line was authenticated using STR profiling (FBI, CODIS) [9]. All of the results are available on the website (http://cellresource.cn). Caco-2 cells were cultured in Dulbecco’s modified Eagle’s medium (DMEM) containing 10% foetal bovine serum (FBS), 1% penicillin-streptomycin, and 1% non-essential-amino acids. Caco-2 cells were maintained at 37 °C in a humidified atmosphere containing 5% CO_2_ and 95% air. The medium was changed every 2 days. The DMEM, FBS, 1% non-essential amino acids, and other materials were purchased from the National Infrastructure of Cell Line Resource (Beijing, China) or Sigma-Aldrich (St. Louis, MO, USA).

### Caprylic acid permeability assay through Caco-2 cell monolayers

For bile acid reabsorption experiments, referring to the report of Y. Wang, et al. [10], cells were seeded and grown on Millicell Hanging Cell Culture Inserts (0.4 μm, Millipore, Molsheim, France) for 21 days to obtain confluent and highly differentiated cell monolayers. The formation of functional epithelial monolayers was monitored via measurement of the transepithelial electrical resistance (TEER) using a Millicell-ERS meter (Millipore) prior to experiments. Cellular morphology was observed using an electron microscope, and permeability was determined using Lucifer Yellow (Sigma, MO, USA) as a marker.

The preparation of fatty acids and CA was performed as described by X. Zhang, et al. [11]. Fatty acids and CA were measured and dissolved in ethanol, then were diluted with cell culture medium containing 20 mg/L endotoxin-free BSA to a final concentration of 50, 100 or 200 μmol/L (ethanol< 0.1%). The dissolved fatty acids and CA were incubated in a water bath at 37 °C for 1 h prior to addition to the cells. Caprylic acid (C8:0), capric acid (C10:0), stearic acid (C18:0) and oleic acid (C18:1) were purchased from Sigma-Aldrich (St. Louis, MO, USA).

**Fig. 1:**
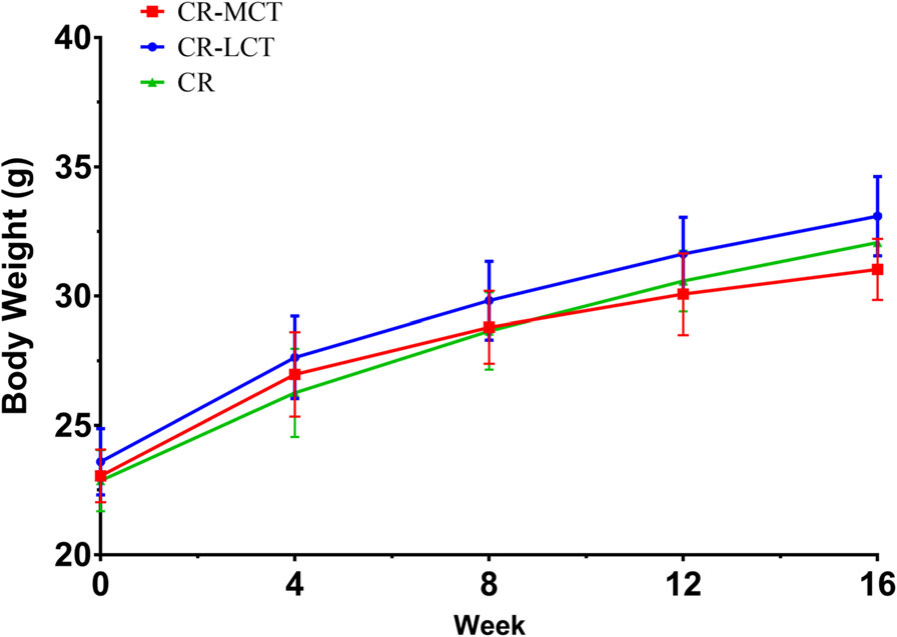
Cell viability of Caco-2 cells in different conditions. Cell viability percentage = OD in experiment group /OD in control group× 100%

The apical (AP) and basolateral (BL) sides of the monolayer were washed with Hank’s Balanced Salt Solution (HBSS) and equilibrated in serum-free complete medium for 15 min. The medium in the AP was replaced with fresh medium-containing CA (200 μmmol/L) or mixed with one of the fatty acids (C8:0, C10:0, C18:0 or C18:1) at 50, 100 or 200 μmol/L. Cell viability of Caco-2 cells in different conditions was assessed by the WST-1 cell cytotoxicity assay (Fig. 1). The medium with one of the fatty acids was defined as the “C8:0, C10:0, C18:0 or C18:1 group”. The “control group” contained no fatty acids, and the “blank group” lacked CA and fatty acids. Only fresh medium was used in the BL. The media from the AP and BL sides were removed 2 h later and stored for analyses of CA using HPLC-MS. The apparent permeability (*P*app) was calculated using the following equation: *P*app = d*Q*/d*t*× 1/*C*_0_*A* (d*Q* is the compound appearance in the receiver compartment, d*t* is the time, *C*_0_ is the concentration in the donor compartment, and *A* is the surface area of the insert).

### I-BABP expression levels in Caco-2 cells

Caco-2 cells were seeded into a 6-well plate (Corning, NY, USA) and cultured to total confluence with high differentiation to confirm the effects of MCFAs on I-BABP [12]. The culture medium was replaced prior to experiments with medium containing delipidized FBS for 24 h. Cells were washed with ice-cold phosphate-buffered saline (PBS) and incubated with fresh medium containing CA at 200 μmol/L (defined as CA group) or CA mixed with one of the fatty acids (C8:0, C10:0, C18:0 or C18:1) at 200 μmol/L (defined as the “C8:0+CA, C10:0+CA, C18:0+CA or C18:1+CA group”) or C8:0 or C10:0 at 200 μmol/L without CA (defined as “C8:0 group and C10:0 group”) for 24 h. And the blank group was without fatty acids and CA. The medium was removed, and cells were washed three times with ice-cold PBS. Total RNA and protein were isolated and collected for further analyses of I-BABP mRNA and protein expression levels.

### Statistical analysis

All data are expressed as the mean ± SD. Data were analysed using one-way analysis of variance followed by the independent t-test to determine the significance of difference between groups using SPSS software version 17.0. *P* < 0.05 was considered statistically significant.

## Results

### Effects of MCT on body weight and blood lipid profiles

**Fig. 2:**
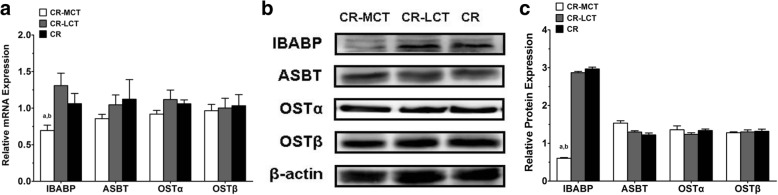
Body weights in C57BL/6J mice during 16 weeks (*n* = 12). ^a^*P* < 0.05, versus CR-LCT group; ^b^*P* < 0.05, versus CR group

No significant differences in body weight were observed between the CR-MCT, CR-LCT and CR groups prior to the experiments. The body weight of the CR-MCT group was significantly lower than that of the CR-LCT group after 12 weeks. There were no differences in body weight between the CR-MCT and CR groups until the 16th week (Fig. 2). Serum TC and LDL-C levels in the CR-MCT group were significantly lower than those in the CR-LCT and CR groups at the end of the study, and the HDL-C/LDL-C ratio was significantly higher in the CR-MCT group than in the CR-LCT group (Table 4).

**Table 4:** Blood lipid profiles in C57BL/6 J mice (*n* = 12)

Blood lipid profiles	CR-MCT	CR-LCT	CR
TC (mmol/L)	3.52 ± 0.65^a,b^	4.13 ± 0.49	4.01 ± 0.33
TG (mmol/L)	0.70 ± 0.29	0.88 ± 0.24	0.86 ± 0.15
HDL- C (mmol/L)	3.12 ± 0.57	3.34 ± 0.39	3.21 ± 0.25
LDL-C (mmol/L)	0.43 ± 0.11^a,b^	0.66 ± 0.18	0.61 ± 0.24
HDL-C/LDL-C	7.54 ± 1.74^a^	5.44 ± 1.61	6.17 ± 2.65
TBA (mmol/L)	16.23 ± 6.51	17.71 ± 5.84	18.23 ± 11.91

^a^*P* < 0.05, versus CR-LCT group

^b^*P* < 0.05, versus CR group

### Effects of MCT on bile acid profiles in bile and faeces

The concentrations of bile acids, including CA, CDCA, LCA, UDCA and TLCA, in the faeces of the CR-MCT group were significantly higher than those in the other groups. TCDCA in the CR-MCT group was higher than that in the CR-LCT group. The excretions of total primary bile acids (CA, CDCA, TCA, TCDCA, GCA and GCDCA), secondary bile acids (DCA, LCA, UDCA, TLCA, TDCA and GDCA) and total bile acids were significantly different in the CR-MCT group compared to the other groups (Table 5).

**Table 5:** Bile acid profiles in faeces in C57BL/6 J mice after 16 weeks (*n* = 5)

BAs in Faeces (mg/3 d)	CR-MCT	CR-LCT	CR
CA	115.11 ± 14.05^a,b^	69.48 ± 11.14	78.03 ± 12.82
CDCA	10.62 ± 1.44^a,b^	4.57 ± 1.01	4.52 ± 0.96
TCA	2.43 ± 0.45	2.7 ± 0.31	2.84 ± 0.43
TCDCA	3.77 ± 0.27^a^	3.05 ± 0.06	3.24 ± 0.98
GCA	1.05 ± 0.27	1.16 ± 0.33	1.33 ± 0.32
GCDCA	7.00 ± 2.92	4.94 ± 2.76	5.38 ± 2.25
Total Primary BAs	139.98 ± 11.44^a,b^	85.89 ± 10.7	95.33 ± 13.89
DCA	2.1 ± 0.22	1.98 ± 0.13	1.62 ± 0.42
LCA	8.71 ± 0.9^a.b^	4.15 ± 0.69	4.55 ± 0.19
UDCA	6.89 ± 0.99^a.b^	5.37 ± 0.73	4.9 ± 0.89
TLCA	0.76 ± 0.02^a.b^	0.48 ± 0.13	0.49 ± 0.05
TDCA	3.82 ± 0.28	3.70 ± 0.14	3.26 ± 0.48
GDCA	1.38 ± 0.45	1.18 ± 0.01	1.48 ± 0.35
Total Secondary BAs	23.66 ± 1.53^a,b^	16.87 ± 1.37	16.3 ± 1.67
Total BAs	163.64 ± 11.6^a,b^	102.77 ± 11.14	111.62 ± 13.96

^a^*P* < 0.05, versus CR-LCT group

^b^*P* < 0.05, versus CR group

There were significant differences in the excretion of CA, CDCA, TCDCA and GCDCA in bile between the CR-MCT group and the other groups. The excretions of total primary bile acids and total bile acids in the CR-MCT group were higher than those in the CR and CR-LCT groups (Table 6).

**Table 6:** Bile acid profiles in bile in C57BL/6 J mice after 16 weeks (*n* = 5)

BAs in bile (mmol/L)	CR-MCT	CR-LCT	CR
CA	92.00 ± 16.05^a,b^	71.42 ± 8.00	62.29 ± 6.70
CDCA	3.65 ± 0.48^a,b^	2.38 ± 0.27	2.50 ± 0.66
TCA	1.53 ± 0.22	1.43 ± 1.00	1.73 ± 0.62
TCDCA	2.97 ± 0.77^a,b^	2.04 ± 0.38	1.85 ± 0.25
GCA	1.75 ± 1.05	1.70 ± 0.57	1.52 ± 0.10
GCDCA	0.56 ± 0.07^a,b^	0.23 ± 0.05	0.25 ± 0.03
Total Primary BAs	102.47 ± 17.51^a,b^	79.19 ± 6.75	70.13 ± 7.51
DCA	0.67 ± 0.09	0.71 ± 0.04	0.66 ± 0.13
LCA	2.03 ± 0.11	1.90 ± 0.28	1.97 ± 0.10
UDCA	0.30 ± 0.05	0.27 ± 0.04	0.27 ± 0.05
TLCA	0.12 ± 0.02	0.14 ± 0.02	0.14 ± 0.02
TDCA	0.13 ± 0.04	0.11 ± 0.03	0.10 ± 0.03
GDCA	0.89 ± 0.13	0.95 ± 0.22	0.97 ± 0.18
Total Secondary BAs	4.14 ± 0.20	4.09 ± 0.44	4.11 ± 0.24
Total BAs	106.61 ± 17.68^a,b^	83.28 ± 6.67	74.25 ± 7.39

^a^*P* < 0.05, versus CR-LCT group

^b^*P* < 0.05, versus CR group

### Effects of MCT on the mRNA and protein expression of bile acid transporters in the small intestine

**Fig. 3:**
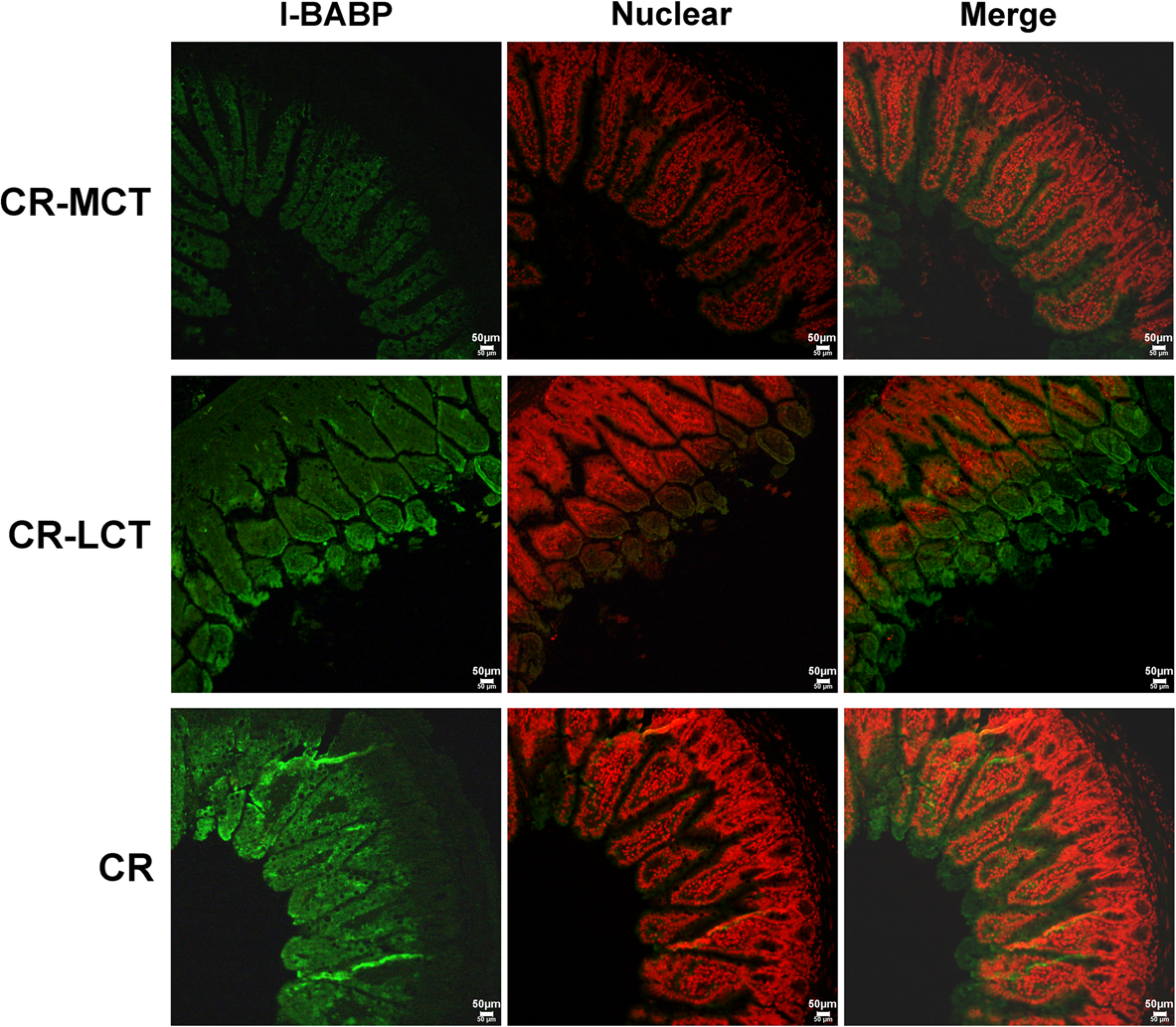
The mRNA and protein expression of bile acid absorption transporters in C57BL/6J mice (*n* = 5). Total RNA and total protein were extracted from small intestine tissues. mRNA transcription levels were measured using real-time PCR analysis, and protein expression levels were measured using Western blot analysis. The housekeeping gene β-actin was used to normalize expression levels. Critical threshold (Ct) values were compared in **a**, while relative light density values were compared in **c**. ^a^*P* < 0.05 versus CR-LCT group; ^b^*P* < 0.05 versus CR group; **a**. critical threshold; **b**. section of blots; **c**. grey-scale analysis

The mRNA expression levels of I-BABP in the small intestine of C57BL/6 J mice in the CR-MCT groups was lower than that in the other groups. However, the transcription levels of ASBT and Ostα/β in the small intestine were not different between groups (Fig. 1). Similar results were observed in the protein expression levels of I-BABP, ASBT and Ostα/β in the small intestine using Western blot analysis (Fig. 3).

**Fig. 4:**
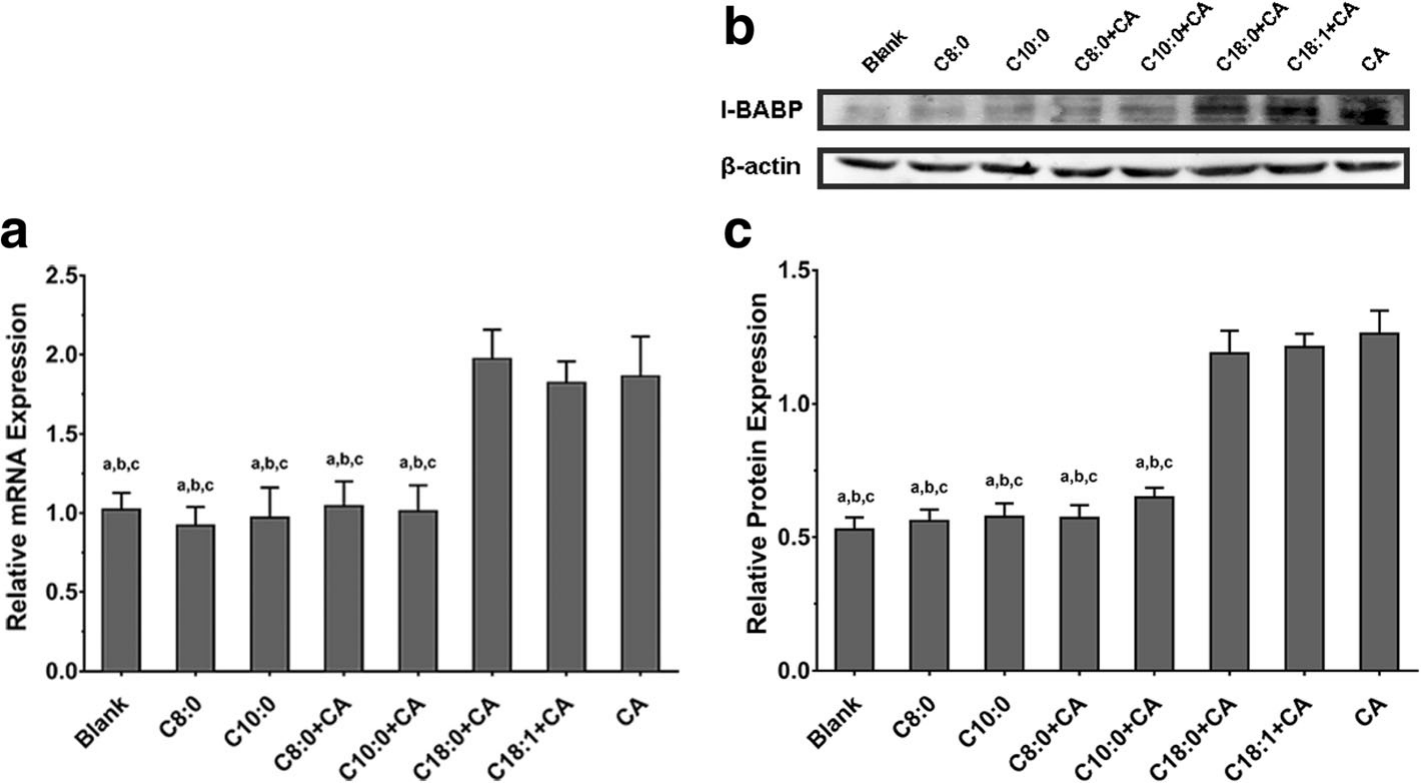
Immunofluorescence of I-BABP in the small intestine in C57BL/6J mice. The bar represents 50 μm in the image. I-BABP was observed as green fluorescence, and the nucleus was observed as red fluorescence

The I-BABP expression level detected by immunofluorescence in the CR-MCT group decreased significantly compared to the other groups. There were no significant change in the I-BABP expression levels in the CR-LCT group and CR group (Fig. 4).

### Effects of MCFAs on cholic acid permeability across Caco-2 cell monolayers

There was no significant difference in the TEER value of Caco-2 monolayer cell model in different experimental conditions. All of TEER values were greater than 500 Ω·cm^2^, which was in line with the the integrity requirements of Caco-2 cell monolayers [13] (Table 7).

**Table 7:** The *P*app values of CA across Caco-2 monolayers treated with various fatty acids and the TEER values (*n* = 5)

Group		C8:0	C10:0	C18:0	C18:1	Control
Condition						
Vehicle		+	+	+	+	+
CA		+	+	+	+	+
C8:0		+	–	–	–	–
C10:0		–	+	–	–	–
C18:0		–	–	+	–	–
C18:1		–	–	–	+	–
Fatty acid concentration						
0 μmol/L	*P*_app_^e^	–	–	–	–	1.16 ± 0.14
	TEER^f^					622.80 ± 56.03
50 μmol/L	*P*_app_^e^	0.87 ± 0.06^a,c,d^	0.93 ± 0.13^a,c^	1.26 ± 0.19	1.06 ± 0.02	–
	TEER^f^	669.80 ± 44.56	713.60 ± 87.06	653.40 ± 59.08	696.60 ± 116.57	
100 μmol/L	*P*_app_^e^	0.74 ± 0.03^a,c,d^	0.81 ± 0.06^a,c,d^	1.06 ± 0.21	1.1 ± 0.09	–
	TEER^f^	710.60 ± 115.01	673.80 ± 60.20	628.40 ± 58.92	668.00 ± 124.00	
200 μmol/L	*P*_app_^e^	0.43 ± 0.07^a,b,c,d^	0.64 ± 0.04^a,c,d^	1.11 ± 0.16	1.02 ± 0.09	–
	TEER^f^	707.00 ± 134.62	591.20 ± 47.56	651.00 ± 57.72	586.20 ± 53.60	

^a^*P* < 0.05, versus control group

^b^*P* < 0.05, versus C10:0

^c^*P* < 0.05, versus C18:0 group

^d^*P* < 0.05, versus C18:1 group

^e^*P*app of CA (AP → BL, cm/s × 10–6)

^f^TEER values of Caco-2 monolayers (Ω·cm^2^)

The *P*app values (apical-to-basolateral) of CA across Caco-2 cell monolayers were significantly lower in the C8:0 and C10:0 groups than in the LCFA groups (C18:0 group and C18:1 group) and control group for the three fatty acid concentrations (except the C10:0 group compared with C18:1 group at 50 μmol/L). This change was more obvious with increased fatty acid concentration. The effect of C8:0 was more significant than that of C10:0 at 200 μmol/L (Table 7).

### Effects of MCFAs on the I-BABP mRNA and protein expression in Caco-2 cells

**Fig. 5:**
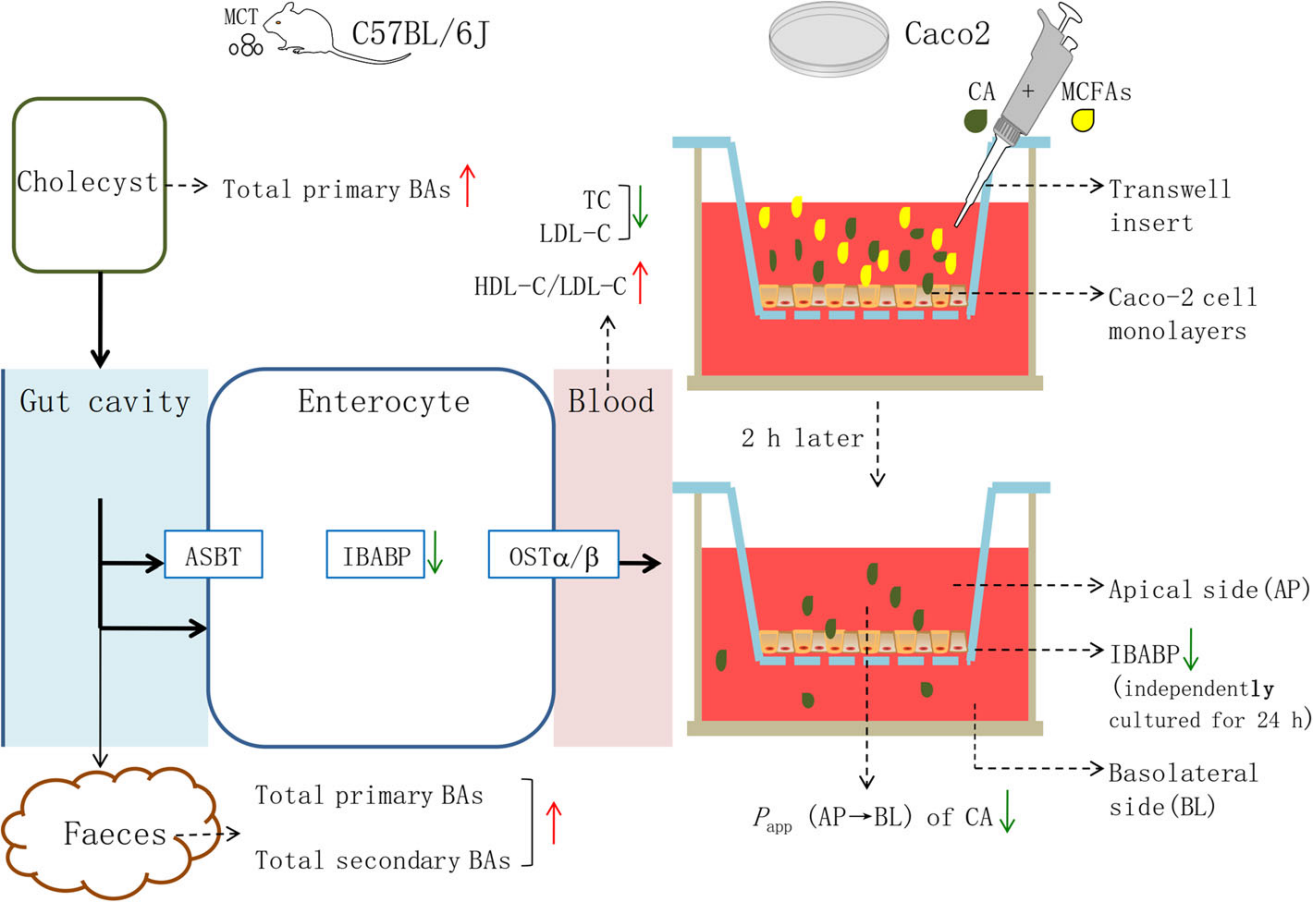
The mRNA and protein expression of I-BABP in Caco-2 cells (n = 5). Total RNA and total protein were extracted from Caco-2 cells, the mRNA transcription level was measured using real-time PCR analysis, and the protein expression level was measured using Western blot analysis. The housekeeping gene β-actin was used to normalize the expression levels, and Critical threshold (Ct) values were compared in **a**, while relative light density values were compared in **c**. ^a^*P* < 0.05 versus CA group; ^b^*P* < 0.05 versus C18:0 + CA; ^c^*P* < 0.05 versus C18:1 + CA group; **a**. critical threshold; **b**. section of blots; **c**. grey-scale analysis

RT-PCR and Western blot analyses demonstrated that (Fig. 5) the expression level of I-BABP in Caco-2 cells treated with MCFAs or with CA (those were C8:0 group, C10:0 group, C8:0 + CA group and C10:0 + CA group) were much lower than the level in those treated with LCFAs with CA (those were C18:0 + CA group, C18:1 + CA group) and CA group.

## Discussion

MCFAs are primarily composed of glycerides of caprylic acids (C8:0) and capric acids (C10:0). Some previous investigations reported that MCTs/MCFAs were beneficial in lipid metabolism and some diseases (e.g., CVDs and Alzheimer’s disease) [14–19]. MCTs in our study slowed the increase in body weight and altered serum lipid profiles (lower levels of TC and LDL-C in the CR-MCT group) in hypercholesterolemic C57BL/6 J mice. The results of serum lipid profiles (lower levels of TC and LDL-C) were consistent with other previous studies [4, 5, 7, 17] which were in different experiment conditions, such as diet formula (MCT or MCFAs with cholesterol-rich diet or high-fat diet) and animal models (obesity mice or hypercholesterolemic mice). Increased serum. TC is a main risk factor of atherosclerosis. HDL-C is regarded as “good cholesterol”, and LDL-C is labelled “bad cholesterol” [1, 20]. MCT may be a protective agent for the cardiovascular system. The absence of significant differences in weight changes in the CR-MCT and CR groups prior to the 12th week might be associated with the composition of the experimental diets in which oil (MCTs/LCTs) was added to basal diets, leading to different levels of total energy between groups. Addition of MCTs did not result in a weight increase even though the energy contributed from fats was increased. Compared to MCFAs, MCTs exhibit more stable physical and chemical properties, particularly a stable fatty acid composition at room temperature. The previous experimental diet required refrigeration because of the volatility and instability of fatty acids. As a promising functional ingredient in food and pharmacy industry applications [21, 22], MCTs are more conveniently stored and provide a more stable nutrient content, which is practical for future applications.

Primary bile acids are converted from cholesterol into CA and CDCA in the liver and combined with taurine and glycine to form binding primary bile acids, which enter into bile. Gallbladder contraction allows the primary bile acids to enter the small intestines and form secondary bile acids in the upper part of the ileum and colon (DCA, LCA and UDCA) [3]. Enterohepatic circulation is essential for the maintenance of cholesterol homeostasis. The faecal excretion of bile acids is a major pathway for the elimination of excessive cholesterol [23]. HPLC-MS was used in our study to evaluate the differences in bile acids in the bile and faeces. The excretion of total primary and secondary bile acids in faeces after treatment was significantly higher in the CR-MCT group. While increased production of bile acids in the liver can lead to increased reabsorption in the small intestine [24], our previous data and this work show that MCT not only promoted bile acid synthesis in the liver [4, 5] but also reduce bile acid reabsorption in the intestine, thus facilitating removal of excess cholesterol from the body.

We specifically focused on the bile acid transport system, which involves the absorption of bile acids into intestinal epithelial cells, to better understand the mechanisms underlying bile acid uptake in the small intestine. Bile acids are reabsorbed from the apical side in the terminal ileum via ASBT. Bile acids enter enterocytes and bind to I-BABP for transport to the basal plane and absorption into the vein via Ostα/β [25]. I-BABP in mice fed an MCT diet exhibited a significant decrease, but no significant changes in ASBT or Ostα/β expression were found. MCTs reduced bile acid reabsorption in the ileum, which partially explains the difference in the content of bile acids between stool and bile. The transport of intracellular bile acids in the enterocytes was mediated via cytosolic I-BABP, which is the bile acid cytoplasmic transporter in the fatty acid binding protein (FABP) family with a similar structure [26]. I-BABP combines with the ASBT transporter to promote bile acid uptake and transported bile acids into the basilar membrane in the cytoplasm. Therefore, I-BABP may be the important protein involved in the transport of bile acids through intestinal epithelial cells [27–29]. The increase of bile acids (primarily CA) in the faeces in the present study may be due to the decreased expression of the related transporter (I-BABP) in the cytoplasm. The lack of change in apical ASBT or basal Ostα/β may partially explain the lack of alterations in serum total bile acids, although faecal bile acids increased. I-BABP may play a critical role in the regulation of bile acid resorption as a buffering agent in the cytoplasm.

We preliminarily hypothesized that MCTs may reduce bile acid reabsorption in the small intestine partially via inhibition of I-BABP expression. We performed a permeability assay of bile acids through Caco-2 cell monolayers for further confirmation. The Caco-2 cell model is an effective tool to investigate the absorption process of orally administered medicines or nutrients [30]. C8:0 and C10:0 decreased the apparent permeability (*P*app) of CA (which was highly excreted in mice fed with MCTs) in the medium (from AP to BL) compared to C18:0 and C18:1 (the highest content of long-chain saturated fatty acids and long-chain unsaturated fatty acids in the experimental diet). The reduction in CA permeation in Caco-2 cell monolayers treated with MCFAs indicated that MCFAs likely decreased bile acid reabsorption in the human small intestine. We investigated whether MCFAs affected I-BABP expression to examine the mechanism of this phenomenon. Caco-2 cells were cultured with CA-mixed fatty acids for analysis. MCFAs (C8:0 or C10:0) with or without CA downregulated I-BABP expression, and CA alone or with LCFAs (C18:0 or C18:1) greatly induced I-BABP expression. The increase in bile acids, which are the biliary factors responsible for the induction of I-BABP expression, upregulated I-BABP expression in a time- and dose-dependent manner [12, 31]. Then, more bile acids were reabsorbed into the small intestine. CA induced I-BABP expression levels in our study. However, this change was not observed when a mixture of CA and MCFAs was used. The same result was seen in MCFAs without CA. It implied MCT might directly affect I-BABP expression to partially reduce the transcellular permeation of bile acids in Caco-2 cells.

The bile acid transport system performs various regulatory mechanisms [32, 33]. Farnesoid X receptor (FXR) plays a critical role in enterohepatic circulation of bile acids by regulating bile acid synthesis, biliary bile acid secretion, intestinal bile acid reabsorption and secretion, and bile acid uptake into hepatocytes. In the ileum, bile acids bind to I-BABP, which is highly induced by FXR [34]. Bile acids are excreted into portal circulation by OSTα/β located in the basolateral membrane of enterocytes [35], which is induced FXR either. Meanwhile FXR is inversely correlated to the bile acid synthetase CYP7A1 expression and regulating the bile acid transporter expression in the liver.Thus, we considered whether MCT affect FXR to increase the excretion of fecal bile acids, and reduce the reabsorption of bile acids in small intestine, and inhibit the expression of I-BABP. Further research was needed to clarify the mechanism of MCT lowering cholesterol by bile acid metabolism.

## Conclusion

**Fig. 6:**
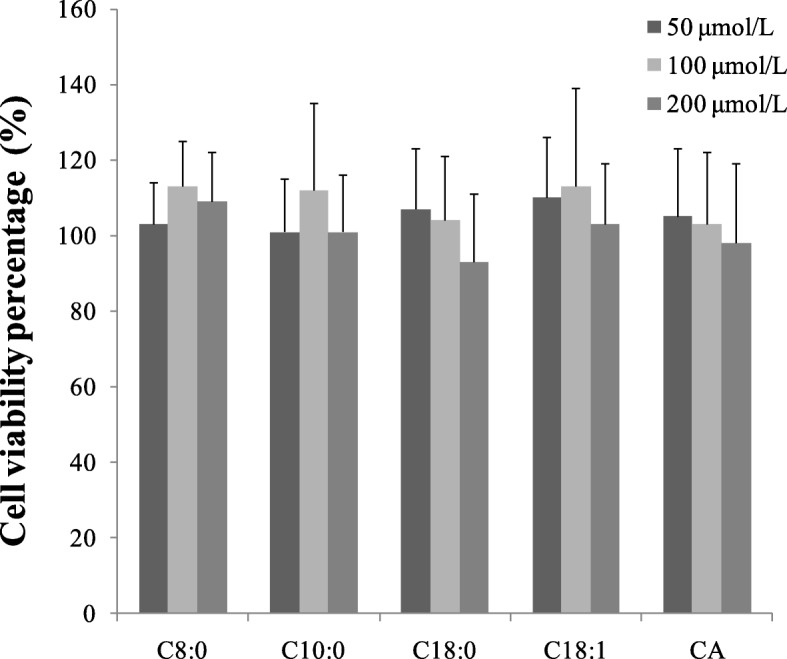
Graphical abstract of the effects of MCTs/MCFAs on reducing intestinal bile acid reabsorption. Arrows (↑) represent an increasing level and upregulation of activity or protein or mRNA expression. Arrows (↓) represent downregulation

MCTs/MCFAs reduce serum cholesterol levels via a partial increase in the excretion of bile acids probably through the reduction of absorption in the small intestines. One possible mechanism is associated with reduced expression of I-BABP (Graphical abstract of the effects of MCTs/MCFAs on reducing intestinal bile acid reabsorption was shown in Fig. 6). MCTs/MCFAs may exert potential therapeutic and protective roles in this process, but the regulatory mechanism is not clear.

## Abbreviations

AP: Apical side
ASBT: Apical sodium-dependent bile salt transporter
BL: Basolateral side
C10:0: Capric acid
C18:0: Stearic acid
C18:1: Oleic acid
C8:0: Caprylic acid
CA: Cholic acid
CDCA: Chenodeoxycholic acid
CHD: Coronary heart disease
CR: Cholesterol-rich
CR-LCT: Cholesterol-rich and long-chain triglyceride
CR-MCT: Cholesterol-rich and medium-chain triglyceride
Ct: Critical threshold
CVDs: Cardiovascular diseases
DCA: Deoxycholic acid
DMEM: Dulbecco’s modified Eagle’s medium
ESI: Electrospray ionization
FABP6: Atty acid binding protein 6
FBS: Foetal bovine serum
FITC: Fluorescein isothiocyanate
GCA: Glycocholic acid
GCDCA: Glycochenodeoxycholic acid
GDCA: Glycodeoxycholic acid
HBSS: Hank’s Balanced Salt Solution
HDL-C: High density lipoprotein-cholesterol
HPLC-MS: High performance liquid chromatography-mass spectrometry
I-BABP: Ileal bile acid binding protein
LCA: Lithocholicacid
LCFAs: Long-chain fatty acids
LCTs: Long-chain triglycerides
LDL-C: Low-density lipoprotein cholesterol
MCFAs: Medium-chain fatty acids
MCTs: Medium-chain triglyceride
Ost-a: Organic solute transporter
Ost-β: Protein name: organic solute transporter
*P*app: Apparent permeability
PBS: Phosphate-buffered saline
PI: Propidium iodide
qRT-PCR: Quantitative real-time PCR
Slc51a: Solute carrier family 51, alpha subunit
Slc51b: Solute carrier family 51, beta subunit
TBA: Total bile acid
TBST: Tris buffered saline with tween
TC: Total cholesterol
TCA: Taurocholic acid
TCDCA: Taurochenodeoxycholic acid
TDCA: Taurodeoxycholic acid
TEER: Transepithelial electrical resistance
TG: Triglyceride
TLCA: Taurolithocholic acid
UDCA: Ursodeoxycholic acid
